# Quasi-1D XY antiferromagnet Sr_2_Ni(SeO_3_)_2_Cl_2_ at Sakai-Takahashi phase diagram

**DOI:** 10.1038/s41598-021-94390-3

**Published:** 2021-07-22

**Authors:** E. S. Kozlyakova, A. V. Moskin, P. S. Berdonosov, V. V. Gapontsev, S. V. Streltsov, M. Uhlarz, S. Spachmann, A. ElGhandour, R. Klingeler, A. N. Vasiliev

**Affiliations:** 1 Lomonosov Moscow State University, Moscow, Russia 119991; 2 National University of Science and Technology “MISiS”, Moscow, Russia 119049; 3 Ural Federal University, Ekaterinburg, Russia 620002; 4 Institute of Metal Physics, RAS, Ekaterinburg, Russia 620990; 5 Hochfeld-Magnetlabor Dresden (HLD-EMFL), Helmholtz-Zentrum Dresden-Rossendorf, 01328 Dresden, Germany; 6 Kirchhoff Institute of Physics, Heidelberg University, 69120 Heidelberg, Germany; 7 Centre for Advanced Materials, Heidelberg University, 69120 Heidelberg, Germany

**Keywords:** Condensed-matter physics, Magnetic properties and materials, Quantum fluids and solids

## Abstract

Uniform quasi-one-dimensional integer spin compounds are of interest as a potential realization of the Haldane conjecture of a gapped spin liquid. This phase, however, has to compete with magnetic anisotropy and long-range ordered phases, the implementation of which depends on the ratio of interchain *J*′ and intrachain *J* exchange interactions and both uniaxial *D* and rhombic *E* single-ion anisotropies. Strontium nickel selenite chloride, Sr_2_Ni(SeO_3_)_2_Cl_2_, is a spin-1 chain system which passes through a correlations regime at *T*_max_ ~ 12 K to long-range order at *T*_N_ = 6 K. Under external magnetic field it experiences the sequence of spin-flop at *B*_c1_ = 9.0 T and spin-flip transitions *B*_c2_ = 23.7 T prior to full saturation at *B*_sat_ = 31.0 T. Density functional theory provides values of the main exchange interactions and uniaxial anisotropy which corroborate the experimental findings. The values of *J*′/*J* = 0.083 and *D*/*J* = 0.357 place this compound into a hitherto unoccupied sector of the Sakai-Takahashi phase diagram.

## Introduction

Among the basic concepts stimulating research on strongly correlated electron systems is the Haldane conjecture of a gapped spin liquid implemented in the uniform chains of Heisenberg integer spins^1,2^. Tens of compounds, both organic and inorganic, have been studied to verify this idea and some of them have been identified as belonging to the Haldane sector of the Sakai-Takahashi phase diagram^3^. This representation takes into account the ratios between interchain *J*′ and intrachain *J* exchange interactions and uniaxial *D* single-ion anisotropy^4^. In addition, properties of uniform integer spin chains can be influenced by rhombic single-ion anisotropy *E*^5^ and anisotropy of spin–spin exchange interactions^6^.

In the Sakai-Takahashi phase diagram, *J*′/*J* versus *D*/*J*, the existence of the Haldane phase is limited by the critical value of *J*′/*J* = 0.0162 for the square lattice of adjacent chains^7^. At positive *D*, the Haldane sector is restricted by *D*/*J* = 0.968^8^, beyond which the large-*D*, or quantum paramagnet, phase continues. At negative *D*, the critical point at *D*/*J* = − 0.316 distinguishes the Haldane and Néel phases^9^.

Both the presence of quantum fluctuations—which are quite pronounced for low-dimensional low-spin systems—and of frustration of interchain exchange interactions suppress the Néel phase. On the contrary, the competition between nearest-neighbor and next-nearest-neighbor exchange interactions within the chains restricts the Haldane phase. Basically, the identification of the quantum ground state of a given magnetic system is possible both experimentally and theoretically. The case of a uniform quasi-one-dimensional spin S = 1 system, strontium nickel selenite chloride, Sr_2_Ni(SeO_3_)_2_Cl_2_, illustrates the remarkable correspondence of these two approaches, which places this compound into the yet unoccupied sector of the Sakai-Takahashi phase diagram, i.e. the sector of an easy-plane XY antiferromagnet at *D* < 1.

**Figure 1:**
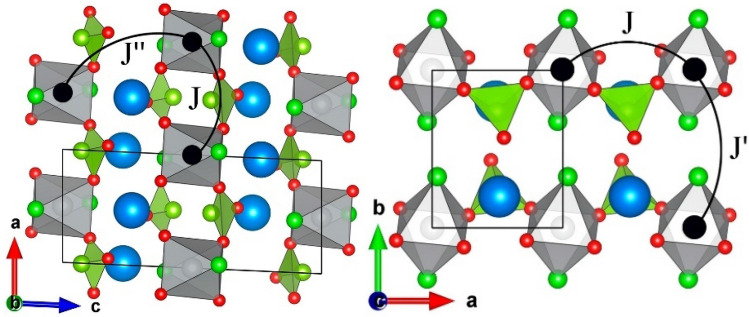
The crystal structure of Sr_2_Ni(SeO_3_)_2_Cl_2_. Strontium ions are shown by blue spheres, nickel ions in black. The solid line marks the unit cell. The arcs mark exchange interaction pathways (see the text). VESTA software has been used for crystal structure visualization^37^.

In the structure of Sr_2_Ni(SeO_3_)_2_Cl_2_, the Ni^2+^ ions are positioned at the center of octahedra formed by four oxygen ions in the basal plane and two chlorine ions in apical positions. The SeO_3_ pyramids bridge the NiO_4_Cl_2_ octahedra into uniform spin *S* = 1 chains running along the *a*-axis. The crystal structure of Sr_2_Ni(SeO_3_)_2_Cl_2_ in polyhedral representation is shown in Fig. 1.

## Results

**Figure 2:**
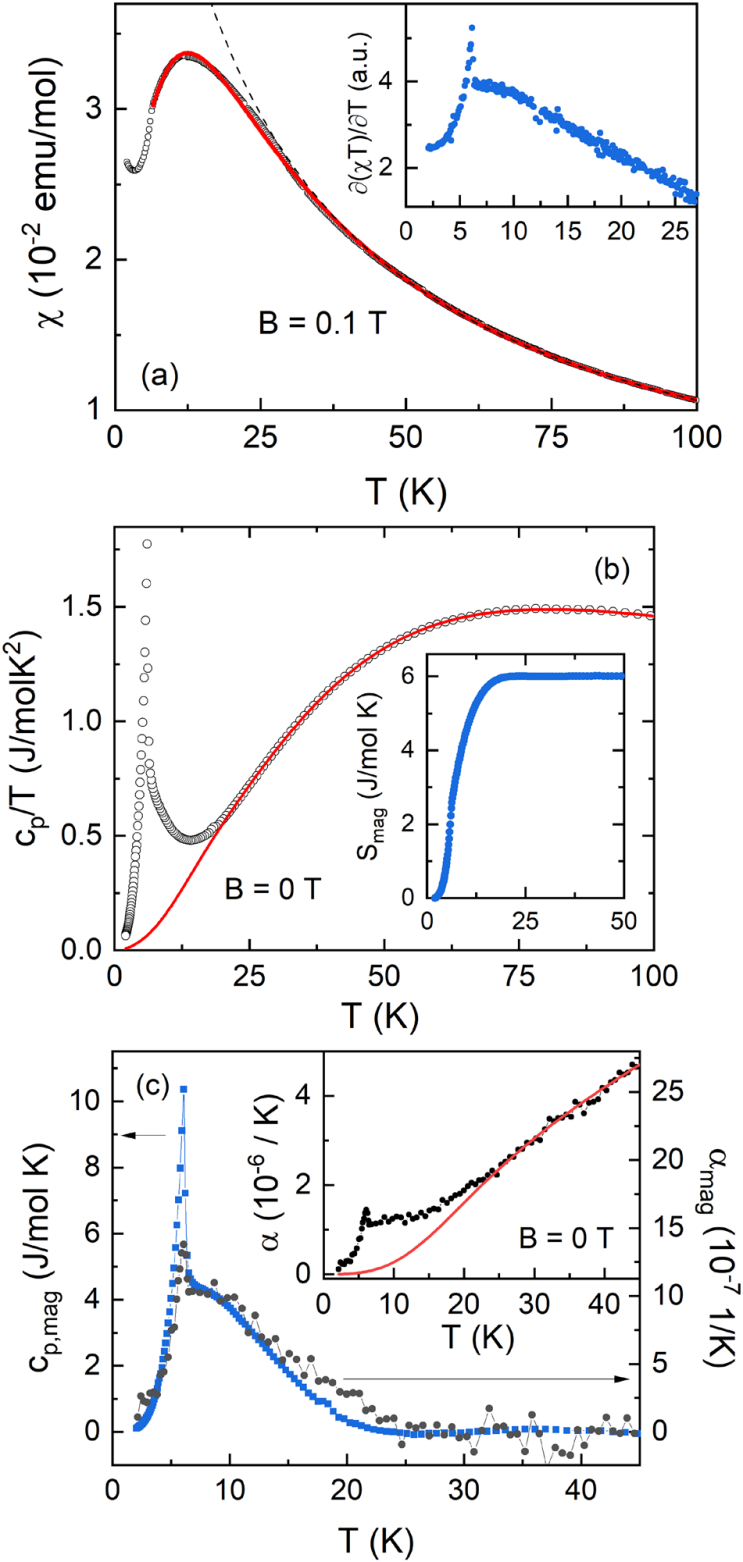
Temperature dependence of the static magnetic susceptibility χ = *M*/*B* taken at *B* = 0.1 T. The solid line represents fit by the Weng Eq. (1). The Fisher specific heat ∂(χ*T*)/∂*T* is shown in the inset (**a**). Temperature dependences of specific heat *c*_p_/*T* (left ordinate) and magnetic entropy *S*_mag_ (right ordinate). The estimated phonon contribution is shown by the solid red line (**b**). Temperature dependences of the magnetic contributions to the specific heat c_p,mag_ and thermal expansion α_mag_. The inset shows α as measured and the phonon background (red line) (c).

The static magnetic susceptibility χ = *M*/*B* reveals the low-dimensional nature of magnetism in Sr_2_Ni(SeO_3_)_2_Cl_2_ as well as its competition with the evolution of long-range magnetic order. Specifically, the magnetic susceptibility demonstrates a Curie–Weiss-like behavior at high temperatures, but shows a pronounced correlation hump at *T*_max_ ~ 12 K before evidencing a kink at the Néel temperature *T*_N_ = 6 K. The formation of long-range magnetic order is most pronounced in the Fisher specific heat ∂(χ*T*)/∂*T*, as shown in the inset to Fig. 2a. The fit by the Curie–Weiss law, i.e. χ = χ_0_ + *C*/(*T* − Θ), gives the Curie constant *C* = 1.277 ± 0.001 emu K/mol thereby defining the effective magnetic moment of the Ni^2+^ ions µ_eff_ = 3.233 ± 0.003 µ_B_ and their g-factor *g* = 2.26 ± 0.01. The Weiss temperature Θ is negative, Θ = − 17.8 ± 0.1 K, which points to the predominance of antiferromagnetic exchange interactions at elevated temperatures. Finally, the temperature independent term χ_0_ = − (1.82 ± 0.02) × 10^–4^ emu/mol equals the sum of Pascal constants of the constituent ions^10^.

The presence of significant antiferromagnetic correlations is evidenced by the fact that the magnetic susceptibility deviates from the Curie–Weiss behavior below about 6*T*_N_. This behavior, including the appearance of a correlation maximum, is typical for a linear spin-1 chain system. As shown in Fig. 2a, in a wide temperature range above *T*_N_ the χ(*T*) dependence is well described by Weng equation^11^

1 $$\frac{\chi {k}_{B}T}{{N}_{A}{(g{\mu }_{B})}^{2}}=\frac{2+0.0194x+0.777{x}^{2}}{3+4.346x+3.232{x}^{2}+5.834{x}^{3}}$$

where $x=\frac{J}{{k}_{B}T}$, *N*_A_, µ_B_ and *k*_B_ are Avogadro, Bohr and Boltzmann constants. For fitting the static susceptibility data (solid line in Fig. 2a), both χ_0_ and the *g*-factor were kept fixed to the abovementioned values resulting in the intrachain exchange interaction parameter *J* = 9.97 ± 0.02 K.

The low-dimensional nature of the spin system in Sr_2_Ni(SeO_3_)_2_Cl_2_ is further corroborated by the specific heat *c*_p_ data in Fig. 2b which in addition to the anomaly at *T*_N_ confirm significant entropy change well above *T*_N_. Describing the lattice specific heat by fitting a model of one Debye and three Einstein modes to the high-temperature regime from 22.5 K up to 200 K (see red line in Fig. 2b) allows obtaining the temperature dependence of the magnetic entropy *S*_mag_, as shown in Fig. 2b (right ordinate). The Debye and Einstein temperatures and respective coefficients obtained from fitting were θ_D_ = 118.3 K, n_D_ = 1.42, θ_E,1_ = 277.4 K, n_E,1_ = 4.55, θ_E,2_ = 635.9 K, n_E,2_ = 3.13, θ_E,3_ = 162.9 K, n_E,3_ = 2.26. The fitting procedure is explained in the methods section. The data imply that about 65% of the full magnetic entropy of *R*ln(3) are released below ~ 25 K while only a portion of which (~ 25%) is spent below *T*_N_. The data thereby point to significant short-range magnetic correlations well above *T*_N_ and hence to the quasi one-dimensional character of magnetism in Sr_2_Ni(SeO_3_)_2_Cl_2_.

The evolution of short-range magnetic correlations is also visible in the thermal expansion coefficient α shown in Fig. 2c. In particular, the data are well described by the phonon background at temperatures up to 55 K, which has been obtained as explained in the methods section, but exhibit a small peak at *T*_N_ and a broad region of non-phononic thermal expansion, thereby implying considerable magneto-elastic coupling in Sr_2_Ni(SeO_3_)_2_Cl_2_. Both the anomaly at *T*_N_ and the length changes in the correlation regime signal the decrease of the volume of the unit cell upon evolution of short-range and long-range magnetic order, respectively. Comparing the thermal expansion coefficients with the specific heat *c*_p_ allows to quantify magneto-elastic coupling by exploiting the Grüneisen ratio γ = α/*c*_p_. As shown in Fig. 2c, the magnetic contributions to the specific heat and to the thermal expansion coefficients can be scaled in the correlation regime. The data hence imply the presence of a single dominating energy scale^12^. As entropy changes are of magnetic nature, we conclude that a single magnetic degree of freedom, i.e., the magnetic intrachain exchange *J*, drives the observed non-phonon length and entropy changes. The corresponding scaling parameter yields the hydrostatic pressure dependence of *J* via the Grüneisen equation^13^ γ = α_mag_/*c*_p,mag_ = 1/*V*_m_ ∂ln*J*/∂*p*|_*T*_ = 0.24(2) mol/MJ, with *V*_m_ = 1.26 10^–4^ m^3^/mol being the molar volume, calculated from the unit cell volume V_cell_ = 417.66 Å^3^^14^. However, the Grüneisen ratio changes at the λ-like anomaly around T_N_ and reaches γ = 0.16(2) mol/MJ. We conclude that the formation of long-range magnetic order at *T*_N_ is either driven by more than one energy scale or an energy different from *J*. This is expected as the evolution of long-range magnetic order appears to result only in the presence of *J*′ and is affected by anisotropy *D*. Quantitatively, using the Ehrenfest relation yields the hydrostatic pressure dependence d*T*_N_/d*p* = *T*_N_*V*_m_γ = 0.12(4) K/GPa.

## Electronic structure and exchange interactions

**Figure 3:**
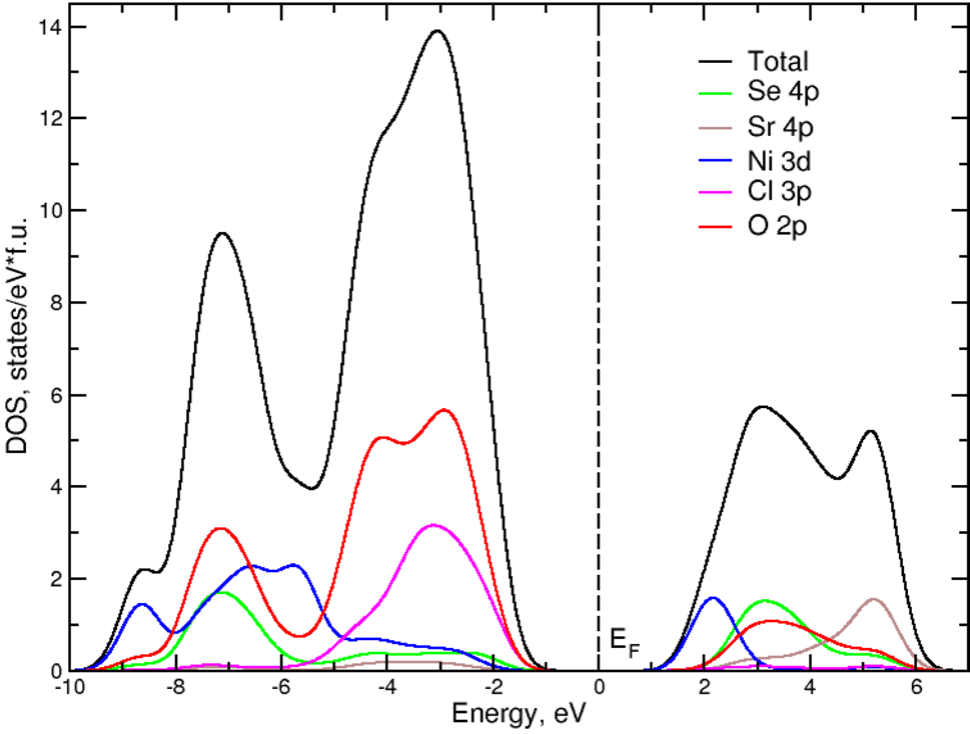
Total and partial density of states (DOS) plots in Sr_2_Ni(SeO_3_)_2_Cl_2_. The Fermi level has been shifted to zero.

The electronic properties including exchange interactions for Sr_2_Ni(SeO_3_)_2_Cl_2_ have been calculated in the frame of the GGA + U approach. Figure 3 represents total and partial densities of states in Sr_2_Ni(SeO_3_)_2_Cl_2_. As in other charge-transfer insulators the top of the valence band is formed mostly by O 2*p* and Cl 3*p* states, while the bottom of the conduction band is dominated by Ni 3*d* states. The band gap is about 2 eV. The valence band width is about 9 eV and there is a strong hybridization of Ni 3*d*, O 2*p* and Cl 3*p* states. The conduction band width is about 5.5 eV.

In order to identify the origin of the low-dimensional magnetic behavior of Sr_2_Ni(SeO_3_)_2_Cl_2_ the calculations of Heisenberg exchange parameters using the total energy method implemented in the JaSS code^15^ have been performed. To obtain the values of the main exchange parameters, shown by arcs in Fig. 1, DFT + U calculations for the supercell, containing 8 Ni^2+^ ions of 4 magnetic configurations, were carried out. The Heisenberg model was chosen to be

2 $$H = \Sigma_{{{\text{i}} > {\text{j}}}} JS_{{\text{i}}} S_{{\text{j}}} + \Sigma_{{{\text{i}} > {\text{k}}}} J^{\prime } S_{{\text{i}}} S_{{\text{k}}} + \Sigma_{{\text{i}}} D\left( {S_{i}^{{\text{z}}} } \right)^{{2}} ,$$

with *J* > 0 corresponding to antiferromagnetic exchange. The sum runs once over each pair of magnetic ions, numerated by *i*,*j* indices for intrachain interactions and *i*,*k* indices for interchain interactions. The calculations yield *J* = 9.75 K—the intrachain exchange interaction parameter between Ni^2+^ ions located at a distance of 5.325(1) Å along the *a***-**axis, *J*′ = 0.8 K—exchange interaction parameter at a distance of 6.436(1) Å along the *b* axis, and *J″* = − 0.35 K—exchange interaction parameter corresponding to a distance of 7.294(1) Å in the *ac*-plane. At these parameters, one might expect a long-range magnetic order with Néel temperature

3 $${T}_{N}\propto \mathit{ln}\left|\frac{{J}^{^{\prime}}}{{J}_{c}^{^{\prime}}}\right|\sqrt{\frac{{J}^{^{\prime}}}{J}}J$$

where critical value of interchain interaction ${J}_{c}^{^{\prime}}\sim {Je}^{-\pi }$ which promotes the formation of true long-range order^16^. |*J*′|+ 2|*J″*|= 1.5 K yields *T*_N_ ~ 4.8 K. The single-ion anisotropy may influence this value. Calculating in the GGA + U + SOC approximation the total energies of different configurations with spins lying in the (oxygen) basal plane and along Ni-Cl bonds we find that the single ion anisotropy is of easy-plane type, *D* = 3.5 K.

## Discussion

**Figure 4:**
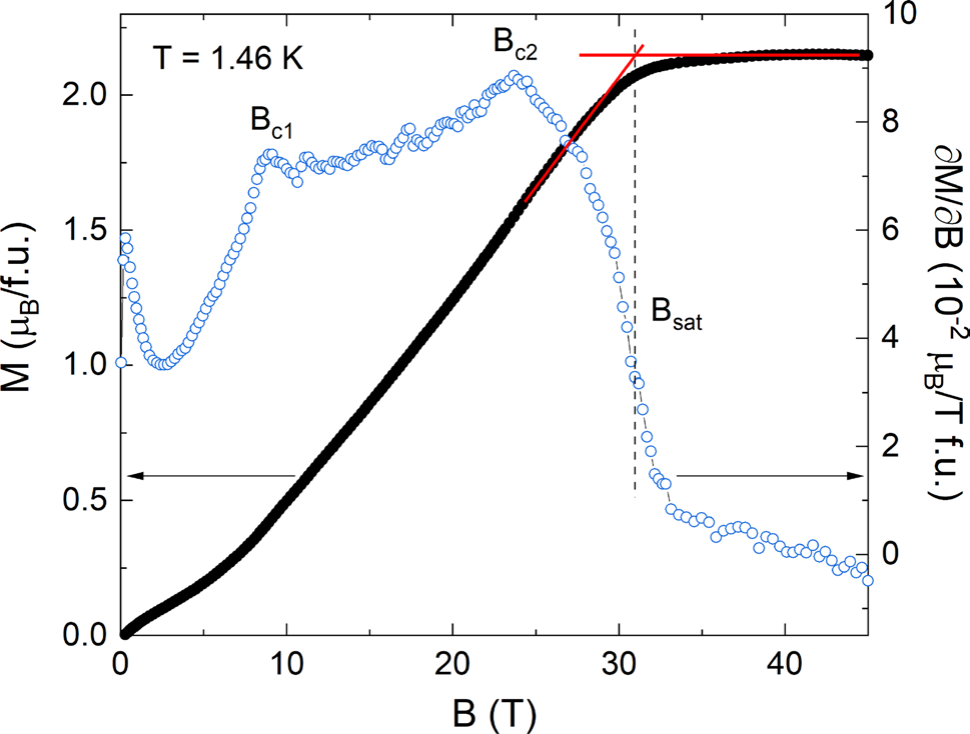
Magnetic field dependence of the magnetization *M* (black filled markers) in the upsweep mode and the magnetic susceptibility ∂*M*/∂*B* (blue open markers) obtained in pulsed magnetic fields up to 45 T at *T* = 1.46 K. The data have been normalized by quasi-static measurements up to 14 T. No appreciable magnetocaloric effect has been noticed. The sequence of critical fields at *B*_c1_, *B*_c2_ and *B*_sat_ can be treated as follows^17^.

The analysis of the static magnetic susceptibility, the specific heat and the thermal expansion clearly confirms the one-dimensional nature of magnetism in Sr_2_Ni(SeO_3_)_2_Cl_2_ governing the properties above *T*_N_ and a separated regime of long-range magnetic order at lowest temperatures. The numerical results agree with the one-dimensional nature of magnetism in this compound. The numerically obtained nearest-neighbor intrachain coupling agrees quite well with the experimentally determined value. Further information on the magnetic parameters is provided by studies of the field dependence of the magnetization, at low temperatures, as shown in Fig. 4. The behavior at lowest magnetic fields, i.e. the convex curvature, is influenced by the presence of about 1% of defects/impurities. At higher fields, the *M*(*B*) curve evidences a sequence of phase transitions at *B*_C1_ = 9 T and *B*_C2_ = 23.7 T while at *B*_sat_ = 31.0 T the magnetization arrives to the full saturation at *M*_sat_ = 2.15 µ_B_.

While the polycrystalline nature of the material under study may suggest the presence of two features at high magnetic fields associated with the saturation fields for the different directions of the field with respect to the *g*-factor, the difference in *B*_c2_ and *B*_sat_ is too large for a reasonable anisotropy of the *g*-factor. In addition, the presence of a low-field feature is unexpected for an easy-plane magnet. The results shown in Fig. 4, however, clearly rule out an easy-axis antiferromagnet where in case of not too large anisotropy the sequence of a spin-flop (at *B*_c1_) and a spin-flip transition (at *B*_c2_) fully describe the evolution of magnetization under magnetic field. Evidently, this is not the case for Sr_2_Ni(SeO_3_)_2_Cl_2_ as the magnetization rises significantly till saturation at *B*_sat_ > *B*_c2_. In contrast, for an easy-plane antiferromagnet there should be no spin-flop transition, since for any direction of external magnetic field the magnetic moments will orient perpendicular to the field. We hence conclude the presence of rhombic anisotropy *E* within the *XY* plane along with uniaxial anisotropy *D* along the *Z* axis in order to account for the observed features. In this case, the sequence of features at *B*_c1_, *B*_c2_ and *B*_sat_ can be treated as follows.

The spin-flop transition within the *XY* plane takes place at

4 $${B}_{c1}=\sqrt{2EJ},$$

the spin-flip transition within the *XY* plane occurs at

5 $${B}_{c2}=2J,$$

and, finally, the full saturation along the *Z* axis takes place at

6 $${B}_{sat}=2J+D.$$

Experimentally found critical values in the *M*(*B*) curve (in Tesla) can be put into correspondence with DFT results (in Kelvin) at the field of the spin-flip transition^17^

7 $$g{\mu }_{B}{B}_{c2}=2S{k}_{B}J$$

The analysis of the *M* versus *B* curve hence yields an additional set of magnetic parameters which not only agree to the value of *J* deduced from the analysis of the static susceptibility, but also agrees well to *J* and *D* obtained numerically from DFT. Both experimental and calculated values of *J*, *D* and *E*, as well as critical fields *B*_c1_, *B*_c2_ and *B*_sat_ are given in Table 1.

**Table 1:** The magnetic subsystem parameters and critical fields in Sr_2_Ni(SeO_3_)_2_Cl_2_. The uncertainties in experimental data are assumed to be one unit in the last quoted digit.

Parameter	Exchange interaction *J*, K	Uniaxial anisotropy *D*, K	Rhombic anisotropy *E*, K	Critical field *B*_c1_, T	Critical field *B*_c2_, T	Critical field *B*_sat_, T
Experimental	9.02 (M vs. B) 9.97 (χ vs. T)	4.86	0.60	9.0	23.7	31.0
Calculated	9.75	3.48			25.7	30.3

**Figure 5:**
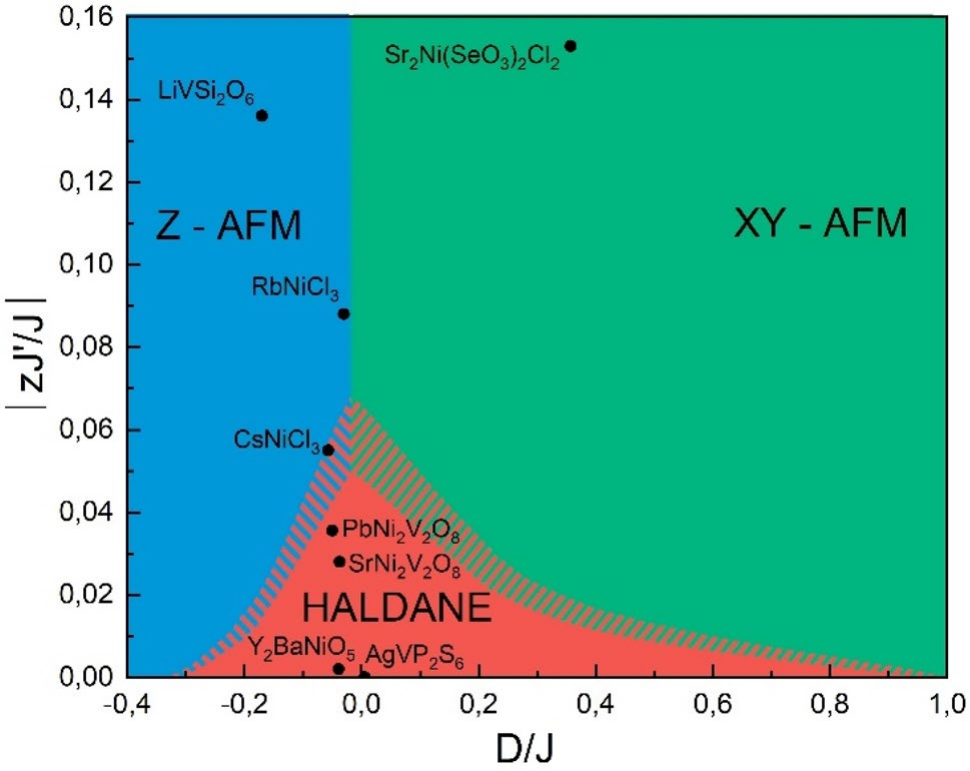
Sakai-Takahashi phase diagram for uniform spin-1 chain compounds. Sharp borders between various phases are obtained within the mean-field approximation, while the comb follows from the quantum Monte Carlo calculations.

In the original calculations of Sakai and Takahashi within the mean-field approximation (MFA) the critical value limiting the Haldane phase at *D* = 0 is *J*′/*J* = 0.013 for the square lattice of adjacent chains and 0.017 for the triangular or kagome lattices^4^. Quantum Monte Carlo calculations^7,8^ provide slightly higher values 0.0162 and 0.0219 for the square and triangular lattices, respectively. As soon as the critical ratio *J*′/*J* depends on the geometry of adjacent chains, for accurate comparison of the systems with different geometry it is preferable to use the quantity z*J*′/*J*, where *z* is the chain coordination number, or more complex equations if different interchain exchange interactions are present. A lower bound for z*J*′/*J* is 0.051 in MFA and 0.068 in QMC^8^. In Sr_2_Ni(SeO_3_)_2_Cl_2_, we find (*J*′ + 2* J″*)/*J* = 0.154 and *D*/*J* = 0.54 (exp) and *D*/*J* = 0.36 (DFT) which place this system into a yet unexplored and rather unique position in the Sakai-Takahashi phase diagram, as shown in Fig. 5.

## Conclusion

The Haldane conjecture on gapped integer spin liquids belongs to the milestones of low-dimensional quantum magnetism^18^. Up to date, several inorganic compounds, i.e. Y_2_BaNiO_5_^19^, AgVP_2_S_6_^20^, PbNi_2_V_2_O_8_^21^ and after some controversy SrNi_2_V_2_O_8_^22,23^, were put into the Haldane sector of the Sakai-Takahashi phase diagram. Few more, CsNiCl_3_^24^, RbNiCl_3_^25^ and LiVSi_2_O_6_^26^ were assigned to the easy-axis antiferromagnetic sector (Z—AFM) of this diagram. Contradictory results have been reported for two trirutile-type tetragonal compounds, NiTa_2_O_6_^27^ and NiSb_2_O_6_^28^. Judging from the claimed values of *J*′/*J* and *D*/*J* ratios both systems should fall into the Haldane sector of this diagram. However, opposite to these estimations both nickel tantalate and nickel antimonite reach long-range order at high enough temperatures *T*_N_ = 10.3 K and 6.7 K, respectively. The title compound, Sr_2_Ni(SeO_3_)_2_Cl_2_, is unequivocally positioned at *J*′/*J* > 0 and 0 < *D*/*J* < 1 in the hitherto unoccupied easy-plane antiferromagnetic (XY—AFM) long-range ordered sector of the Sakai-Takahashi phase diagram. This makes Sr_2_Ni(SeO_3_)_2_Cl_2_ an extremely interesting compound both for further theoretical and experimental studies. Varying the alkaline-earth metal or applying external pressure or strain one obtains a playground for studying yet unexplored region of the phase diagram and gaining deeper insight into Haldane physics.

## Methods

The sample of strontium nickel selenite chloride was obtained from SrSeO_3_ and anhydrous NiCl_2_ precursors^29^. The mixture of 0.2 g SrSeO_3_ and 0.06 g of NiCl_2_ has been ground in the agate mortar and loaded into a quartz tube. The tube was sealed under vacuum and placed into the furnace for 10 days at 750 °C. The X-ray pattern was fully indexed in the monoclinic unit cell with parameters *a* = 5.3373(21)Å, *b* = 6.4526(23) Å, *c* = 12.231(4) Å, β = 92.518(20)°, space group *P*2_1_/*n*^30^.

Thermodynamic properties, i.e. magnetization *M* and specific heat *C*_p_, of the pressed pellet sample were studied using various options of Magnetic Properties Measurements System MPMS-7 T and Physical Properties Measurements System PPMS-14 T (Quantum Design) up to 14 T in the temperature range from 2 to 300 K. Pulsed-magnetic-field magnetization has been measured up to 45 T using a coaxial pick-up coil system. The pulsed-field magnetization data have been calibrated by means of static magnetic field measurements. The measurements of thermal expansion have been provided by means of a three-terminal high-resolution capacitance dilatometer in a home-built set-up^31^. The final fitting procedure, after trying several ways of approaching the fitting, for the phonon background was the following: first, the thermal expansion data up to 55 K, excluding the range from 3 to 22.5 K was fitted with one Debye and two Einstein modes. This yielded θ_D_ = 118.3 K, θ_E,1_ = 277.4 K and θ_E,2_ = 1497.81 K. θ_E,2_ only contributes to the overall thermal expansion above 70 K. Therefore, the thermal expansion data was re-fit with one Debye mode and one Einstein mode with fixed θ_D_ = 118.3 K, θ_E_ = 277.4 K, which corresponds to the background shown in Fig. 2 and used to obtain α_mag_. To satisfactorily describe the specific heat data, two Einstein modes were then added and fit with fixed θ_D_ = 118.3 K, θ_E,1_ = 277.4 K and freely varying θ_E,2_ and θ_E,3_. This fit resulted in the final parameters as described in the text. Lastly, the fit was subtracted from *c*_p_ to obtain the magnetic contribution *c*_p,mag_ to the specific heat.

To calculate the electronic and magnetic structure of Sr_2_Ni(SeO_3_)_2_Cl_2_ within the density functional theory the pseudopotential VASP code^32^ with the Perdew-Burke-Ernzerhof version of the exchange correlation potential^33^ has been used. To take into account the strong electronic correlations on Ni the GGA + U approach has been applied^34^. On-site Hubbard U and intra-atomic exchange J_H_ for Ni^2+^ ions were chosen to be 8.0 and 0.9 eV, respectively^35,36^. A 5 × 3 × 3 k-mesh in the symmetry-irreducible part of the first Brillouin zone was used in all calculations. The convergence condition for the total energy was set to 10^–6^ eV.

## Data Availability

The datasets generated and/or analyzed during this study are available to qualified requestors from the corresponding author.
